# A High-Density Genetic Map of an Allohexaploid *Brassica* Doubled Haploid Population Reveals Quantitative Trait Loci for Pollen Viability and Fertility

**DOI:** 10.3389/fpls.2018.01161

**Published:** 2018-08-28

**Authors:** Su Yang, Sheng Chen, Kangni Zhang, Lan Li, Yuling Yin, Rafaqat A. Gill, Guijun Yan, Jinling Meng, Wallace A. Cowling, Weijun Zhou

**Affiliations:** ^1^Institute of Crop Science and Zhejiang Key Laboratory of Crop Germplasm, Zhejiang University, Hangzhou, China; ^2^The UWA Institute of Agriculture, The University of Western Australia, Perth, WA, Australia; ^3^Institute of Digital Agriculture, Zhejiang Academy of Agricultural Sciences, Hangzhou, China; ^4^UWA School of Agriculture and Environment, Faculty of Science, The University of Western Australia, Perth, WA, Australia; ^5^National Key Laboratory of Crop Genetic Improvement, Huazhong Agricultural University, Wuhan, China; ^6^School of Biological Sciences, Faculty of Science, The University of Western Australia, Perth, WA, Australia

**Keywords:** restriction-site associated DNA sequencing, allohexaploid *Brassica*, single nucleotide polymorphism, loss of chromosomes, collinearity, QTL mapping

## Abstract

A doubled haploid (DH) mapping population was obtained from microspore culture of an allohexaploid F_1_ from the cross between two recently-synthesized allohexaploid *Brassica* lines. We used single nucleotide polymorphism (SNP) genetic variation based on restriction-site associated DNA (RAD) sequencing to construct a high density genetic linkage map of the population. RAD libraries were constructed from the genomic DNA of both parents and 146 DH progenies. A total of 2.87 G reads with an average sequencing depth of 2.59 × were obtained in the parents and of 1.41 × in the progeny. A total of 290,422 SNPs were identified from clustering of RAD reads, from which we developed 7,950 high quality SNP markers that segregated normally (1:1) in the population. The linkage map contained all 27 chromosomes from the parental A, B and C genomes with a total genetic distance of 5725.19 cM and an average of 0.75 cM between adjacent markers. Genetic distance on non-integrated linkage groups was 1534.23 cM, or 21% of total genetic distance. Out of 146 DH progenies, 91 had a complete set of 27 chromosomes as expected of a hexaploid species, and 21 out of 27 chromosomes showed high collinearity between the physical and linkage maps. The loss of chromosome(s) or chromosome segment(s) in the DH population was associated with a reduction in pollen viability. Twenty-five additive QTL were associated with pollen viability and fertility-related traits (seed number, seed yield, pod length, plant height, 1000-seed weight). In addition, 44 intra-genomic and 18 inter-genomic epistatic QTL pairs were detected for 4 phenotypic traits. This provides confidence that the DH population may be selected for improved pollen viability and fertility in a future allohexaploid *Brassica* species.

## Introduction

*Brassica* species, including vegetable and oilseed crops, are of major importance in agricultural production around the globe. Six agriculturally important *Brassica* species form a group known as the U's triangle (U, 1935). Three allotetraploid species (*Brassica juncea*, AABB; *B. napus*, AACC; and *B. carinata*, BBCC) were derived from interspecific hybridization between three diploid species (*B. rapa*, AA; *B. nigra*, BB; and *B. oleracea*, CC) (U, 1935). A new hexaploid *Brassica* based on species in U's triangle may help to expand the geographical range, yield and quality of *Brassica* crops, based on the success of allohexaploidy in wheat and other crops (Chen et al., 2011).

No naturally occurring allohexaploid species with all three genomes in U's triangle (2*n* = AABBCC) are known to exist (Chen et al., 2011). Recently, some allohexaploid *Brassica* materials were synthesized through several different interspecific hybridization approaches (Chen et al., 2011; Gupta et al., 2016). Allohexaploid *Brassica* were produced through interspecific crosses between *B. rapa* and *B. carinata, B*. *nigra* and *B*. *napus*, and crosses among the allotetraploid species *B. juncea, B. napus* and *B. carinata* (Pradhan et al., 2010; Geng et al., 2013). Such allohexaploids may contain beneficial alleles and enhanced genetic diversity derived from each of the *Brassica* “U's Triangle” species, with the added benefit of hybrid vigor that may be conferred by pairing alleles from different species, for example, C-genome alleles derived from *B. napus* and *B. oleracea* may be paired in the synthetic allohexaploid species. Several allohexaploid hybrids were used to create doubled haploid (DH) populations of hexaploid *Brassica* (Geng et al., 2013). Among them, hybrid H16-1 and its synthetic allohexaploid *Brassica* parents were all hexaploid (6x) with 2*n* = 54 chromosomes. Since H16-1 was derived from 4 different *Brassica* species, the paternal and maternal chromosomes in H16-1 showed high allelic diversity. In addition, H16-1 was one of the two most prolific hybrids in production of DH progenies by microspore culture (Geng et al., 2013). The DH population derived from hybrid H16-1 was used to create the first framework genetic map of allohexaploid *Brassica* based on simple sequence repeat markers (Yang et al., 2016b).

Next generation sequencing (NGS) has the capability of capturing millions of DNA short reads (50–100 bp) in a short time with relatively low cost. Restriction-site associated DNA (RAD) sequencing (RAD-seq) is one of the most effective methods based on NGS technology with restriction digestion for SNP discovery (Davey et al., 2011). SNPs are considered as the most abundant and widely distributed genetic markers throughout the genome (Hillier et al., 2008; Brunner et al., 2009; Davey et al., 2011; Hansey et al., 2012). RAD-seq has been successfully applied to genetic map construction in various species, such as *B. napus* (Bus et al., 2012), barley (Chutimanitsakun et al., 2011), cotton (Wang et al., 2012a), lupin (Yang et al., 2012), and stickleback (Baird et al., 2008). However, successful identification and validation of SNPs in polyploid crops, which have large and complex genomes, is relatively difficult since the presence of paralogous loci from duplicated segments of the genome or homoeologous loci from individual sub-genomes (Wang et al., 2012b).

In this research, we present the first high density SNP genetic linkage map of allohexaploid *Brassic*a with SNP markers derived from RAD-seq technology. In addition, seven phenotypic traits including seed number, seed yield, pod length, plant height, 1000-seed weight, pollen viability and seed color were mapped. QTL and epistatic QTL pairs were detected among these seven phenotypic traits.

## Materials and methods

### Mapping materials and DNA extraction

Genetic mapping materials were the same as described in Yang et al. (2016b). Maternal line 7H170-1, derived from several generations of selfing from the cross of *B. carinata* x *B. rapa* (Tian et al., 2010), and paternal line Y54-2, generated from the cross of *B. napus* x *B. nigra* (Pradhan et al., 2010), produced an allohexaploid hybrid H16-1 from which a DH population was created by microspore culture (Geng et al., 2013). After checking the hexaploid DNA content of the DH lines by flow cytometry (Geng et al., 2013), 146 DH lines were chosen as the mapping materials in this study. Young leaf tissues were collected from both parents and 146 DH progenies and DNA was extracted following the CTAB protocol (Doyle, 1987).

### Library construction and RAD-seq

A RAD library for the DH progenies was constructed following the protocol described earlier by Baird et al. (2008) with slight modifications. The genomic DNA (0.5–1.0 mg) from both parents and 146 DH lines were digested with 20 units of EcoR I (GAATTC) at 37°C for 15 min. DNA fragments were ligated with P1 adapters first and then randomly sheared by sonication. Fragments with the length range of 300–500 bp were selected using agarose gel electrophoresis and purified with QIA quick Gel Purification Kit (Qiagen). P2 adapters were ligated to the purified fragments. Libraries with both P1 and P2 adapters were enriched by PCR amplification and RAD sequencing was performed on an Illumina HiSeq 4000 platform at Beijing Genomics Institute (BGI), Shenzhen, China.

### RAD-seq data analysis, SNP calling and genotyping

Firstly, the original data obtained by HiSeq 4000 were transformed into raw data by base calling. Sequence reads without the *Hind*III recognition site were discarded. Sequences reads with uncertain bases among the first 50 bp or with more than 3 uncertain bases among the whole sequences were removed from the reads. Sequence reads that did not conform to Q20 quality control were also excluded. The flanking sequencing of enzyme digestion sequences were clustered to get RAD tags. RAD tags were compared, clustered and indexed to find SNPs among allohexaploid parents and DH progenies. SOAP2 (Li et al., 2009) was used to compare the sequenced reads with reference genome sequence of *B. rapa* (Wang et al., 2011), *B. nigra* (Yang et al., 2016a), *B. oleracea* (Liu et al., 2014). Sequence depth and sequence coverage were calculated with respect to comparing results. SNPs, InDels and SVs were detected by SOAPsnp (Li et al., 2009), SOAPindel (Li et al., 2012), and SOAPsv (Luo et al., 2012). Bayesian model was used to calculate the probability of every possible genotype according to the observed comparison results. The genotype with the highest probability level was considered as the correct genotype.

### Measurement of phenotypic traits

After checking the ploidy level using flow cytometry (Geng et al., 2013), all microspore-derived hexaploid DH individuals were transplanted into potting mix in 10-cm square pots in a growth chamber at 15°C at The University of Western Australia. After 14 days, plants were transferred to the glasshouse with natural light and photoperiod in April-May (late autumn). Diseases and pests were controlled during the growing season and fertilizer applied to optimize plant growth. Pollen viability was measured at the early flowering stage. Three young open flowers were collected and the pollen grains were stained with 1% acetocarmine. Approximately 600 pollen grains were counted. Normal viable pollen grains were large, round and densely stained red and easily distinguished from small, non-stained dead pollen and immature microspore stage cells (Li et al., 1995). Plant height was measured from ground level to the tip of the main inflorescence. Pod length was measured by the average length of five randomly selected pods. Seed was harvested from each plant, and seed number per plant, seed yield per plant, and 1000-seed weight were measured after 7 days of drying in a 32°C oven.

### Linkage map construction and QTL mapping

We performed chi-square goodness-of-fit test on all the SNPs to check whether they conformed to the 1:1 expectation for segregation in a DH population. SNPs that failed to conform to this segregation ratio (*p* < 0.01) or had more than 8% missing genotypes in the population were removed from the linkage analysis. If there were any missing data or no polymorphisms among the parent lines, these SNPs were also excluded from this research.

A linkage map of SNP markers that survived the rigorous filtering process was formed using JoinMap 4.1 (Van Ooijen, 2006) at LOD value between 9 and 30 using Kosambi function to convert recombination frequency to map distance (centiMorgan, cM). We used whole genome sequencing data of *B. rapa* (Wang et al., 2011), *B. nigra* (Yang et al., 2016a), *B. oleracea* (Liu et al., 2014) to identify the chromosome positions of SNPs. Those markers which were unequivocally assigned to a chromosome were used to identify chromosomes A1–A10 (from the *B. rapa* sequence), B1–B8 (from the *B. nigra* sequence), and C1–C9 (from the *B. oleracea* sequence). Finally, 274 SSRs from the linkage map of Yang et al. (2016b) were added to form a consolidated linkage map according to the genetic or physical map position of the SSRs on the genome. The twenty-seven chromosomes in the consolidated linkage map were compared with the previous linkage groups of Yang et al. (2016b).

QTL mapping was performed using QTL IciMapping 4.1 (Wang et al., 2016). The inclusive composite interval mapping (ICIM) method was used to detect QTL. Additive QTL with LOD value less than 1.4 were ruled out to ensure the detection accuracy and reliability. ICIM-ADD method was used to detect A × A QTL with LOD value more than 2.5. Additionally, linear correlation coefficients between the six phenotypic traits were determined by Pearson correlation using SPSS 16.0 (SPSS Inc., USA).

### Collinearity analysis

Collinearity analysis was carried out using sequence information of each mapped marker, which was searched against reference genome sequences of *B. rapa* (Wang et al., 2011), *B. nigra* (Yang et al., 2016a), *B. oleracea* (Liu et al., 2014) using SOAPsnp (Li et al., 2009). To generate the figure, cM distances on the genetic linkage maps were scaled up by a factor of 100,000 to match similar base pair lengths of the chromosomes of reference genomes. Figures were visualized using Circos plotting tool (Krzywinski et al., 2009) in order to identify syntenic regions between the genetic map (genetic position in cM) and physical map (physical position in Mb) of this allohexaploid *Brassica*.

### Statistical analysis

The data were analyzed using a statistical package, SPSS version 16.0 (SPSS, Chicago, IL, USA). The variation among DH lines in infertility, pollen viability and other phenotypic traits was evaluated by one-way analysis of variance (ANOVA) followed by least significant difference test. Results were considered significant at *P* < 0.05.

## Results

### SNP discovery and genotyping

In our study, RAD sequencing was performed on an Illumina HiSeq 4000 platform. A total of 2.87 G reads were obtained with average sequencing depth of 2.59 × in the 2 allohexaploid parents and 1.41 × in the 146 DH progenies. Average length of reads was 82 base pairs (bp). Among these reads, 2.44 G were clean reads including 35.2 M reads in the maternal parent and 25.84 M reads in the paternal parent (Table 1). The number of reads for each DH progeny was 16.68 M on average, ranging from 3.9 to 31.4 M (Table 1). The total number of bp acquired among maternal parent, paternal parent and DH population was 3.38, 2.48, and 233.77 Gbp, respectively (total 239.63 Gbp), with an average number of 1.6 Gbp per DH progeny, ranging from 0.37 to 3.01 Gbp (Table 1). The average GC % and Q20 ratios were 38.93 and 98.53% in the DH progenies. The Q20 value for all individuals was higher than 97.32% (Table 1).

**Table 1:** Summary of data obtained by RAD-seq in allohexaploid *Brassica* parents and doubled haploid progenies.

**Sample**	**Read (M)**	**Base pair (Gbp)**	**GC (%)**	**Q20 (%)**	**Sample**	**Read (M)**	**Base pair (Gbp)**	**GC (%)**	**Q20 (%)**
P1	35.20	3.38	39.69	98.27	SY096	14.14	1.36	38.29	97.50
P2	25.84	2.48	39.83	98.22	SY097	14.83	1.42	39.47	98.63
SY003	15.68	1.51	39.15	98.64	SY098	13.42	1.29	38.04	98.52
SY009	15.59	1.50	38.87	97.48	SY099	18.08	1.74	38.19	98.45
SY010	13.13	1.26	39.40	98.56	SY100	18.96	1.82	38.70	98.69
SY011	16.19	1.55	39.60	98.74	SY101	13.72	1.32	37.94	98.51
SY012	16.39	1.57	38.95	98.56	SY102	14.99	1.44	38.95	98.54
SY014	17.23	1.65	38.93	98.60	SY103	28.66	2.75	38.57	98.56
SY015	22.18	2.13	38.47	98.60	SY104	14.82	1.42	38.20	98.47
SY016	21.57	2.07	38.20	98.48	SY105	17.62	1.69	39.56	98.56
SY017	16.31	1.57	39.05	98.64	SY107	20.02	1.92	37.70	97.47
SY018	15.64	1.50	38.64	98.64	SY108	18.04	1.73	38.49	98.66
SY019	14.62	1.40	38.75	98.60	SY109	18.98	1.82	38.78	98.42
SY020	16.80	1.61	39.30	98.65	SY110	11.81	1.13	38.66	98.61
SY021	25.79	2.48	39.48	98.65	SY112	15.52	1.49	39.50	98.65
SY022	15.56	1.49	39.39	98.72	SY113	15.56	1.49	37.68	98.27
SY024	18.70	1.79	38.94	98.62	SY115	16.43	1.58	38.99	98.44
SY025	16.12	1.55	38.20	97.47	SY116	16.61	1.59	38.89	98.52
SY033	15.49	1.49	39.86	98.73	SY117	13.67	1.31	39.27	98.63
SY034	18.33	1.76	39.06	98.67	SY118	13.14	1.26	39.25	98.65
SY036	18.53	1.78	38.53	98.68	SY119	15.60	1.50	39.39	98.68
SY038	16.35	1.57	38.41	98.70	SY120	23.98	2.30	38.87	98.67
SY040	16.92	1.62	39.18	98.62	SY121	17.43	1.67	39.08	98.68
SY041	12.49	1.20	39.17	98.55	SY122	18.42	1.77	39.41	98.63
SY042	20.82	2.00	38.48	98.70	SY124	16.73	1.61	39.52	98.41
SY043	12.46	1.20	38.11	98.47	SY127	17.73	1.70	38.46	98.57
SY044	17.58	1.69	38.39	98.67	SY129	16.91	1.62	38.25	98.51
SY045	21.74	2.09	38.71	98.60	SY130	13.04	1.25	37.72	97.32
SY046	9.94	0.95	38.26	97.45	SY131	17.54	1.68	38.14	98.56
SY047	13.39	1.29	38.18	97.53	SY132	19.21	1.84	38.50	98.57
SY048	17.36	1.67	38.27	98.69	SY135	15.31	1.47	38.38	98.57
SY049	18.80	1.81	38.55	98.66	SY136	18.31	1.76	38.76	98.59
SY051	13.47	1.29	38.46	98.59	SY138	13.94	1.34	38.19	98.36
SY052	16.68	1.60	38.03	98.47	SY139	20.06	1.93	38.25	98.57
SY053	23.88	2.29	38.52	98.38	SY140	16.84	1.62	38.39	98.57
SY054	13.00	1.25	40.01	98.68	SY141	17.58	1.69	37.97	98.45
SY055	14.54	1.40	38.68	98.53	SY142	14.86	1.43	39.80	98.55
SY056	15.00	1.44	39.69	98.69	SY143	15.54	1.49	39.75	98.54
SY057	16.88	1.62	39.08	98.74	SY144	15.08	1.45	39.86	98.59
SY058	14.12	1.36	38.10	98.53	SY146	14.15	1.36	39.72	98.60
SY059	16.30	1.56	39.50	98.61	SY147	18.52	1.78	39.85	98.60
SY060	18.37	1.76	38.91	98.64	SY148	15.81	1.52	39.47	98.55
SY061	16.15	1.55	39.19	98.65	SY149	13.41	1.29	40.23	98.54
SY062	26.52	2.55	39.36	98.75	SY154	13.61	1.31	39.89	98.31
SY063	12.09	1.16	38.16	98.53	SY155	31.40	3.01	37.76	98.71
SY064	15.57	1.49	39.01	98.60	SY156	17.25	1.66	37.98	98.72
SY065	19.46	1.87	38.76	98.55	SY160	14.99	1.44	39.39	98.74
SY066	18.94	1.82	39.54	98.77	SY162	17.16	1.65	38.09	98.75
SY067	26.71	2.56	37.87	98.52	SY163	16.70	1.60	38.24	98.72
SY069	12.62	1.21	39.02	98.52	SY164	10.52	1.01	37.82	98.58
SY071	15.76	1.51	40.29	98.72	SY165	12.94	1.24	38.58	98.51
SY072	12.69	1.22	39.18	98.57	SY166	17.77	1.71	38.22	98.75
SY073	18.53	1.78	38.33	98.46	SY167	14.59	1.40	39.69	98.72
SY074	18.32	1.76	39.82	98.72	SY168	15.26	1.46	38.44	98.74
SY075	12.76	1.22	40.16	98.72	SY169	17.73	1.70	38.32	98.72
SY076	21.49	2.06	39.95	98.64	SY170	21.43	2.06	38.15	98.52
SY077	16.54	1.59	38.38	98.59	SY171	15.37	1.48	39.12	98.48
SY078	17.41	1.67	39.35	98.76	SY172	16.19	1.55	40.23	98.47
SY079	15.70	1.51	39.72	98.73	SY173	17.80	1.71	39.66	98.50
SY080	20.38	1.96	40.03	98.68	SY174	16.47	1.58	39.91	98.50
SY081	16.79	1.61	39.99	98.44	SY175	19.68	1.89	39.99	98.51
SY082	13.10	1.26	40.33	98.68	SY176	17.79	1.71	39.65	98.50
SY083	20.98	2.01	38.12	97.54	SY177	17.09	1.64	39.68	98.51
SY085	20.66	1.98	38.50	98.68	SY178	12.33	1.18	38.71	98.61
SY086	14.12	1.36	39.13	98.64	SY179	17.04	1.64	39.39	98.29
SY087	19.43	1.87	38.28	98.50	SY180	17.44	1.67	39.74	98.75
SY088	16.24	1.56	38.57	98.66	SY181	11.07	1.06	38.84	98.37
SY089	17.69	1.70	38.44	98.65	SY186	12.62	1.21	38.49	98.47
SY090	16.58	1.59	38.84	98.49	SY187	18.83	1.81	40.57	98.77
SY091	17.76	1.70	38.61	98.68	SY188	10.01	0.96	40.01	98.74
SY092	14.80	1.42	38.25	98.60	SY189	15.21	1.46	38.83	98.39
SY093	13.86	1.33	39.09	98.64	SY190	16.93	1.63	39.99	98.72
SY094	18.36	1.76	38.50	98.70	SY191	19.74	1.89	39.48	98.53
SY095	15.30	1.47	38.24	98.47	SY192	3.90	0.37	39.79	98.71
					Total	2435.14	233.77		
					Average	16.68	1.60	38.93	98.53

*P1 is maternal line 7H170-1, P2 is paternal line Y54-2, while SY003 to SY192 are 146 doubled haploid progenies derived from hybrid H16-1*.

A total of 290,422 RAD markers were used to detect SNPs using the clustering method in parents and DH progeny, and the reference genomes were used to find the exact location of SNPs on chromosomes of the three species. The number of polymorphic SNPs between parents was 238,868. The number of SNPs in DH progenies ranged from 174,629 to 263,339 with an average number of 220,412 (Table S1). The average percentage of homozygous and heterozygous SNP loci among the DH progenies was 93.52 and 6.48%, respectively.

The *B. rapa* (Wang et al., 2011), *B. nigra* (Yang et al., 2016a), *B. oleracea* (Liu et al., 2014) reference genomes were used to annotate SNPs in the allohexaploid parents and DH progenies. The number of coding sequence SNPs in maternal and paternal parents was 20,833 and 10,262, respectively, and ranged from 71 to 11,572 in the DH progenies, as a result of higher sequencing depth in parents. Among the SNPs in the coding sequences, there were two kinds of SNPs: synonymous (silent) and non-synonymous. The distribution of these two types of SNPs varied in the hexaploid *Brassica* parents and DH progenies (Table S1). In the maternal and paternal parent, there were 12,478 and 6,063 synonymous SNPs, and 8,355 and 3,582 non-synonymous SNPs, respectively. The average number of synonymous and non-synonymous SNPs in 146 DH progenies was 3,002 and 2,020, respectively (Table S1). That is, the number of synonymous SNPs was about 1.5 times of that of non-synonymous SNPs in both parents and DH progeny. Among the non-synonymous SNPs, there were four kinds of large-effect SNPs, including premature stop SNPs, stop codon to non-stop codon SNPs, start codon to non-start codon SNPs and splice site SNPs. The average number per progeny of these four kinds of SNPs was 24.56, 4.46, 2.99, and 15.83, respectively (Table S1). In addition, 14,422 and 7,069 indels (insertion-deletion) with the length of 1–5 bp were discovered in the maternal and paternal parent, respectively. The average number of insertions and deletions in the DH population was 3,121 and 3,065, respectively (Table S1).

Among the 416,238 SNPs identified in the allohexaploid *Brassica* parents and DH progenies, 329,244 SNPs had a missing rate exceeding 8% in DH progenies and were removed. For the remaining 86,994 SNPs, 12,965 were polymorphic in the parents and had no missing data. SNPs belonged to similar sites or deviated seriously from expected segregation ratios (*P* < 0.01) were excluded. Finally, 7,950 SNPs reached Q20 quality limits and were used for linkage map construction (Table S2). In addition, 274 SSR markers from Yang et al. (2016b) were included in linkage map construction.

### Construction and characterization of SNP linkage map

**Figure 1:**
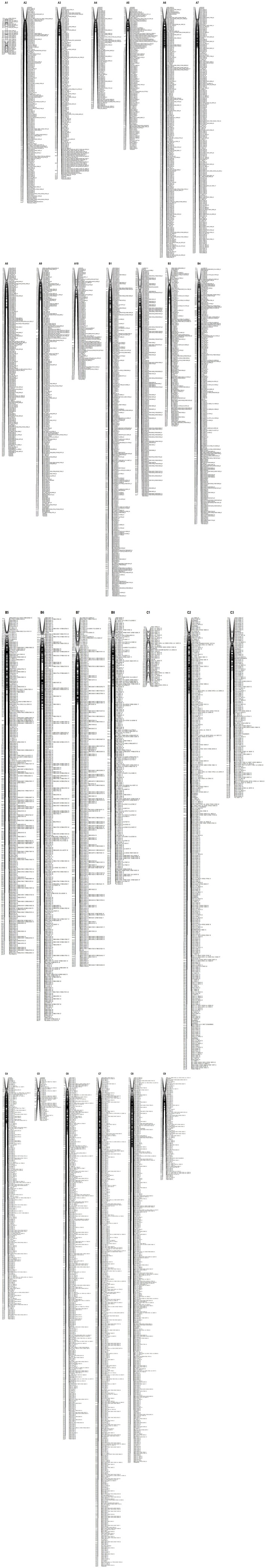
The allohexaploid *Brassica* genetic linkage map formed from a doubled haploid mapping population derived from allohexaploid *Brassica* hybrid H16-1. The map is composed of 27 chromosomes including 10 A genome, 8 B genome and 9 C genome chromosomes with 7,499 SNP markers and spans 5,714.82 cM. The map distances are shown on the left of the chromosome while the names of the SNP markers are indicated on the right.

Among the 7,950 SNPs, 7,499 were successfully integrated into the linkage map while 451 SNP makers failed to be anchored into the linkage map. In addition, 163 out of 274 SSR markers were integrated into the SNP linkage groups according to their physical map position relative to SNPs (Figure 1). The allohexaploid linkage map spanned 5725.19 cM with an average distance of 0.75 cM between adjacent markers on 27 chromosomes, including 10 A genome, 8 B genome, and 9 C genome chromosomes (Figure 1). The total genetic distance of the A genome (1200.67 cM) was about half of that of B (2348.90 cM) and C (2175.63 cM) genomes. The number of mapped SNP and SSR markers per genome followed the same order as genetic distance, with the A genome (1,929) less than the B (2,844) and C (2,889) genomes (Table 2). The number of markers per chromosome varied greatly within genomes (Tables 2, 3, Figure 1). Chromosome C7 had the largest number of markers (698) while chromosome A1 had the least number of markers (30) (Figure 1, Table 2), with an average of 284 markers per chromosome. The average length of the 27 chromosomes was 212.04 cM. The average interval between genetic RAD loci ranged from 0.37 to 5.98 cM across chromosomes.

**Table 2:** The length of chromosomes (cM) and distribution of markers in the genetic linkage map of a doubled haploid population derived from hexaploid *Brassica* hybrid H16-1.

**Chromosome**	**Length of chromosome (cM)**	**No. of markers**	**Max interval (cM)**	**Min interval (cM)**	**Average interval (cM)**
A1	179.29	30	24.02	0.03	5.98
A2	112.94	227	5.14	0.00	0.50
A3	87.87	214	5.38	0.00	0.41
A4	66.76	114	7.04	0.00	0.59
A5	64.83	174	5.67	0.00	0.37
A6	155.11	271	8.54	0.00	0.57
A7	155.83	247	5.81	0.00	0.63
A8	159.43	209	12.16	0.00	0.76
A9	114.96	300	3.37	0.00	0.38
A10	103.65	143	8.92	0.00	0.72
B1	352.91	436	6.96	0.00	0.81
B2	270.21	313	31.42	0.00	0.86
B3	132.92	205	8.98	0.00	0.65
B4	326.94	342	12.09	0.00	0.96
B5	297.87	397	19.52	0.00	0.75
B6	366.62	434	39.7	0.00	0.84
B7	343.45	411	32.11	0.00	0.84
B8	257.98	306	7.53	0.00	0.84
C1	238.88	53	32.46	0.00	4.51
C2	291.67	402	6.07	0.00	0.73
C3	182.36	158	23.11	0.00	1.15
C4	220.22	324	9.03	0.00	0.68
C5	68.92	70	11.7	0.00	0.98
C6	289.56	526	6.5	0.00	0.55
C7	380.21	698	7.29	0.00	0.54
C8	370.87	520	9.66	0.00	0.71
C9	132.94	138	10.97	0.00	0.96
Average	212.04	283.78			
Total	5725.19	7662			

**Table 3:** The distribution of SNPs on each linkage group of a doubled haploid population derived from a hexaploid *Brassica* hybrid H16-1 and the physical map of the reference genome of *B. rapa* (Wang et al., 2011), *B. nigra* (Yang et al., 2016a) and *B. oleracea* (Liu et al., 2014).

**(i)**	**Physical map**									
**Linkage map**	**A01**	**A02**	**A03**	**A04**	**A05**	**A06**	**A07**	**A08**	**A09**	**A10**
A1	14	1	3	1	1	1	0	0	0	0
A2	1	187	0	0	0	0	0	1	0	0
A3	0	0	154	3	1	0	0	0	0	1
A4	0	0	0	96	0	0	0	0	0	1
A5	0	0	0	0	122	1	0	1	1	2
A6	0	0	1	0	0	218	0	0	0	0
A7	0	1	1	0	2	0	216	0	0	0
A8	0	0	0	0	0	0	0	170	0	2
A9	0	0	2	0	0	0	1	3	220	0
A10	0	0	0	0	0	1	1	0	0	120
**(ii)**	**Physical map**									
**Linkage map**	**B01**	**B02**	**B03**	**B04**	**B05**	**B06**	**B07**	**B08**		
B1	7	106	11	3	15	15	24	22		
B2	0	307	0	0	0	0	0	0		
B3	3	25	19	10	0	12	4	20		
B4	4	8	7	8	8	15	100	17		
B5	0	0	6	6	339	0	0	0		
B6	0	0	0	0	0	387	0	0		
B7	0	0	2	1	0	0	380	0		
B8	8	33	11	10	8	0	35	31		
**(iii)**	**Physical map**									
**Linkage map**	**C01**	**C02**	**C03**	**C04**	**C05**	**C06**	**C07**	**C08**	**C09**	
C1	13	7	3	1	6	1	2	1	5	
C2	0	283	4	2	6	2	2	1	1	
C3	0	1	124	2	2	1	0	0	0	
C4	0	0	2	242	1	0	0	1	1	
C5	1	0	0	2	7	1	4	1	1	
C6	3	2	3	0	3	385	5	1	4	
C7	4	4	2	2	0	1	515	0	7	
C8	1	2	0	2	1	0	4	433	3	
C9	0	0	2	1	5	0	2	0	89	

*Common SNPs found in the same order on the (i) A, (ii) B, and (iii) C reference genomes and the genetic map (shaded in yellow), and also noting SNPs which originate from other specific sites on reference genome*.

Of the 451 SNP and 111 SSR markers that were not integrated into the high-density linkage map, 186 were A-genome specific markers, 57 were B-genome specific, 168 were C-genome specific and 151 were markers with unknown position. The 562 non-integrated markers included 66 single unlinked loci and 48 extra linkage groups (XLG), including 3 XLG with more than 50 markers, 11 XLG with 6 to 50 markers, five quintuplets, six quadruplets, seven triplets and 16 duplets covering a total genetic mapping distance of 1534.23 cM (Figure S1).

By blasting the RAD tags with the *B. rapa, B. nigra*, and *B. oleracea* reference genomes, the chromosome position of 84.44% of the 7,499 mapped SNPs was confirmed. Among them, 1,695 SNPs were located in the A reference genome, 2,027 in the B reference genome, and 2,610 in the C reference genome. If the SNPs on the allohexaploid *Brassica* genetic map and the reference genome were on the same chromosome, these were referred to as “common” SNPs. Common SNPs ranged from 1.62 to 99.03% of all SNPs per chromosome, with an average of 65.07%. The percentage of common SNPs was highest in A genome (79.52%), followed by C genome (63.38%), and B genome (48.92%).

### Collinearity analysis

**Figure 2:**
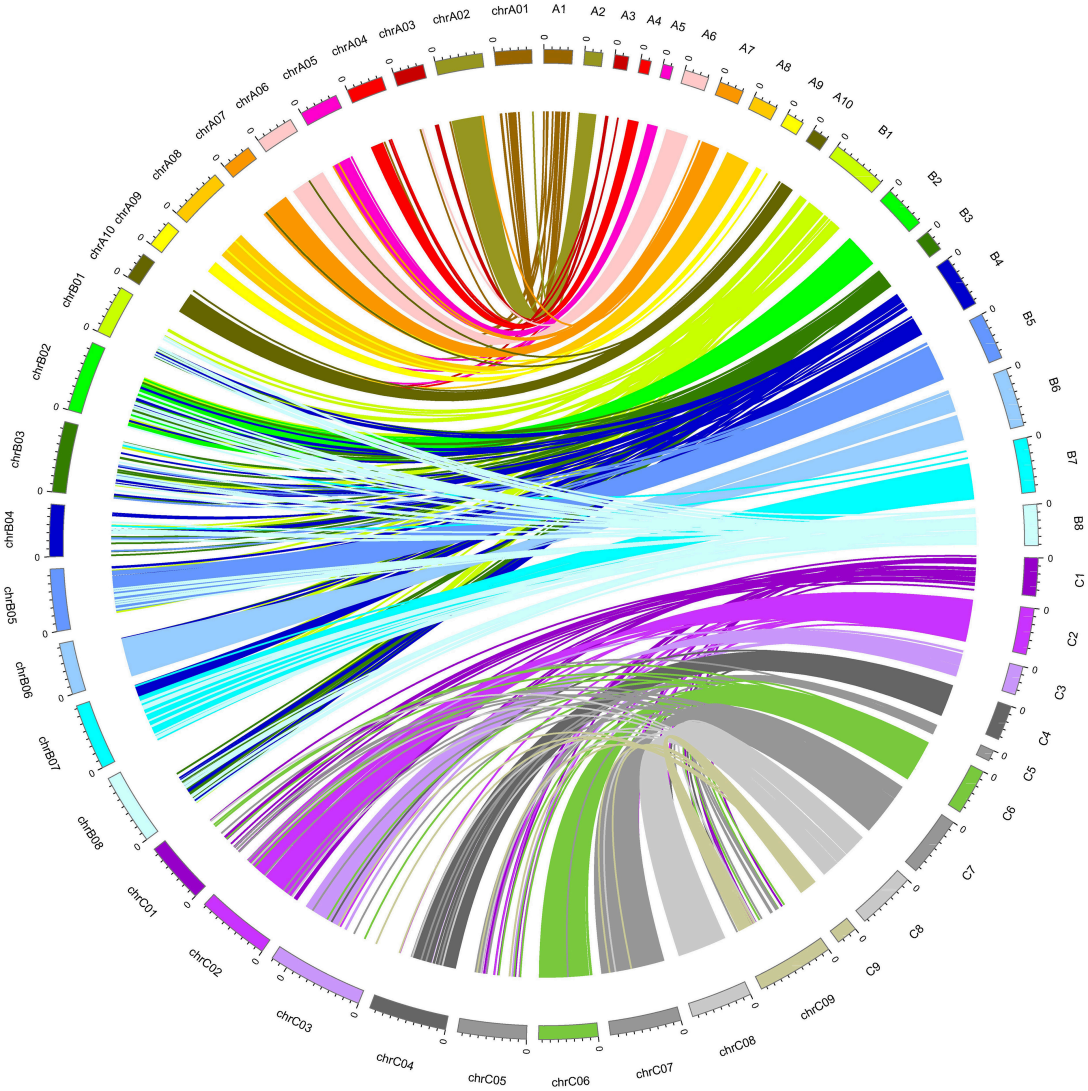
Collinearity between the genetic map (A1–A10, B1–B8, C1–C9) and the physical map (chrA01-chrA10, chrB01-chrB08, chrC01-chrC09).

Collinearity analysis of A-, B-, and C-subgenomes in this hexaploid *Brassica* linkage map shows good collinearity with the reference genome sequence of *B. rapa, B. nigra*, and *B. oleracea* on most A, B, and C chromosomes (Figure 2), and show consistent genetic mapping of common SNPs, especially in the A and C genome. Only 6/27 linkage groups have low rates of common SNPs; the majority appear to be relatively stable (Table 3). We have temporarily nominated these six linkage groups as B1, B3, B4, B8, C1, and C5 (Table 3). There are substantial “introgressions” (contiguous blocks of SNPs) inside these six linkage groups from other chromosomes (Figure 2).

### Chromosome status in the DH population

Out of 146 first generation DH progenies used for linkage mapping, 91 had a complete set of 27 chromosomes as expected of a hexaploid species, 51 had lost one or more chromosomes on the physical map (more than 80% SNPs missing on one or more chromosomes) and another 27 had lost one or more segments (between 20 and 80% of contiguous SNPs missing on one or more chromosomes) (Table S4).

### Pollen viability, fertility and chromosome status

The first-generation DH progeny plants derived from hybrid H16-1 showed a wide range of phenotypic traits including seed yield per plant, 1000-seed weight, seed number per plant, pollen viability, plant height, and pod length. Fertile plants (those which produced at least one seed) were significantly taller and had longer pods than infertile plants (Table 4). However, there was no difference in mean pollen viability between fertile and infertile groups of progeny, indicating that the pollen viability was not associated with the fertility in this hexaploid *Brassica* population (Table 4).

**Table 4:** Population means and standard errors for phenotypic traits scored on 146 doubled haploid progenies derived from hexaploid *Brassica* hybrid H16-1.

**Groups**	**Number of plants**	**Seed yield per plant**	**1000-seed weight**	**Seed number per plant**	**Pollen viability**	**Plant height**	**Pod length**
		**(g)**	**(g)**		**(%)**	**(cm)**	**(mm)**
Fertile	53	0.131 ± 0.029	3.686 ± 0.405	43.7 ± 8.52	53.08 ± 4.19	78.7 ± 2.0	29.1 ± 0.9
Infertile	93	NA	NA	NA	50.41 ± 2.95	65.6 ± 2.5	11.9 ± 1.4
Difference					NS	^***^	^***^

Progeny were divided into fertile (produced seeds) and infertile (produced no seeds). The difference between fertile and infertile progeny for pollen viability, plant height and pod length was evaluated by a t-test for groups with unequal variances. NS, not significantly different;

*** *significantly different at P < 0.001*.

These phenotypic traits were further investigated in relation to loss of chromosome(s) or chromosomal segment(s) in these DH plants. Loss of chromosome(s) or chromosome segment(s) was not associated with fertility, seed yield per plant, 1000-seed weight, seed number per plant, plant height, or pod length, but pollen viability of DH plants with intact chromosomes (Group III, 60.6%) was significantly higher than those with loss of chromosome(s) (Group I, 46.1) and those with loss of chromosome segment(s) (Group II, 41.9%) (Table 5). These results suggest that the loss of chromosome(s) or chromosome segment(s) significantly reduced pollen viability, but did not reduce fertility.

**Table 5:** The average performance of the infertility and 6 agronomic traits among 4 groups with different chromosome status.

		**I**	**II**	**III**	**IV**
**Chromosome group**		**Loss of Chromosome**	**Loss of chromosome segment**	**Intact chromosome**	**Uncertain**
Total number in category		51	27	64	4
Infertility^a^	Number infertile in category	30	18	42	3
	Mean proportion infertile (%)	58.8 a^b^	66.7 a	65.6 a	75.0 a
Seed yield per plant	Number in category	21	9	22	1
	Mean ± sd (g)	0.120 ± 0.160 a	0.143 ± 0.289 a	0.138 ± 0.195 a	0.095
1000-seed weight	Number in category	21	9	22	1
	Mean ± sd (g)	4.049 ± 4.028 a	3.205 ± 1.973 a	3.573 ± 2.251 a	2.879
Seed number per plant	Number in category	21	9	22	1
	Mean ± sd	40.5 ± 51.1 a	37.9 ± 69.8 a	49.6 ± 69.0 a	33.0
Pollen viability	Number in category	43	23	55	3
	Mean ± sd (%)	46.1 ± 26.8 a	41.9 ± 25.0 a	60.6 ± 25.5 b	24.8 ± 21.5 a
Plant height	Number in category	39	23	57	3
	Mean ± sd (cm)	71.7 ± 19.4 a	70.3 ± 19.1 a	69.8 ± 21.0 a	76.0 ± 9.7 a
Pod length	Number in category	28	18	45	2
	Mean ± sd (mm)	26.7 ± 6.7 a	25.1 ± 7.8 a	24.9 ± 7.7 a	31.0 ± 18.4 a

If more than 80% of SNPs are missing on a particular chromosome, this is shown as “loss of chromosome.” If more than 20% but less than 80% of SNPs are missing as a contiguous segment on a chromosome, this is shown as “loss of chromosome segment.”

a There were no differences in infertility among groups according to contingency chi-square test across four groups, chi-square = 0.95 (not significant, df = 3);

b *Group means accompanied by a common letter do not differ according to the least significant difference test at P = 0.05 (group IV not included in analysis where only 1 value)*.

### QTL mapping of pollen viability and fertility traits

Pearson correlation coefficients among different pairs of the 6 agronomic traits ranged from −0.115 to 0.946 (Table 6). Seed yield was positively correlated with seed number (*P* < 0.01) and pod length (*P* < 0.05) (Table 6). Seed number was positively correlated with pollen viability and pod length (*P* < 0.05). Pod length was correlated with plant height (*P* < 0.05). No significant negative correlation was found among these 6 traits (Table 6).

**Table 6:** Pearson correlation coefficients among seed number, seed yield, pod length, plant height, 1000-seed weight and pollen viability measured on 146 doubled haploid progenies derived from a hexaploid *Brassica* hybrid H16-1.

**Phenotypic trait**	**Seed yield**	**1000-seed weight**	**Seed number**	**Pollen viability**	**Plant height**	**Pod length**
Seed yield	1	-0.018	0.946^**^	0.173	0.0760	0.398^*^
1000-seed weight		1	−0.169	−0.153	0.024	0.023
Seed number			1	0.205^*^	0.075	0.433^*^
Pollen viability				1	−0.115	0.080
Plant height					1	0.430^*^
Pod length						1

** and

* *represent the significance levels of 1 and 5%, respectively*.

**Figure 3:**
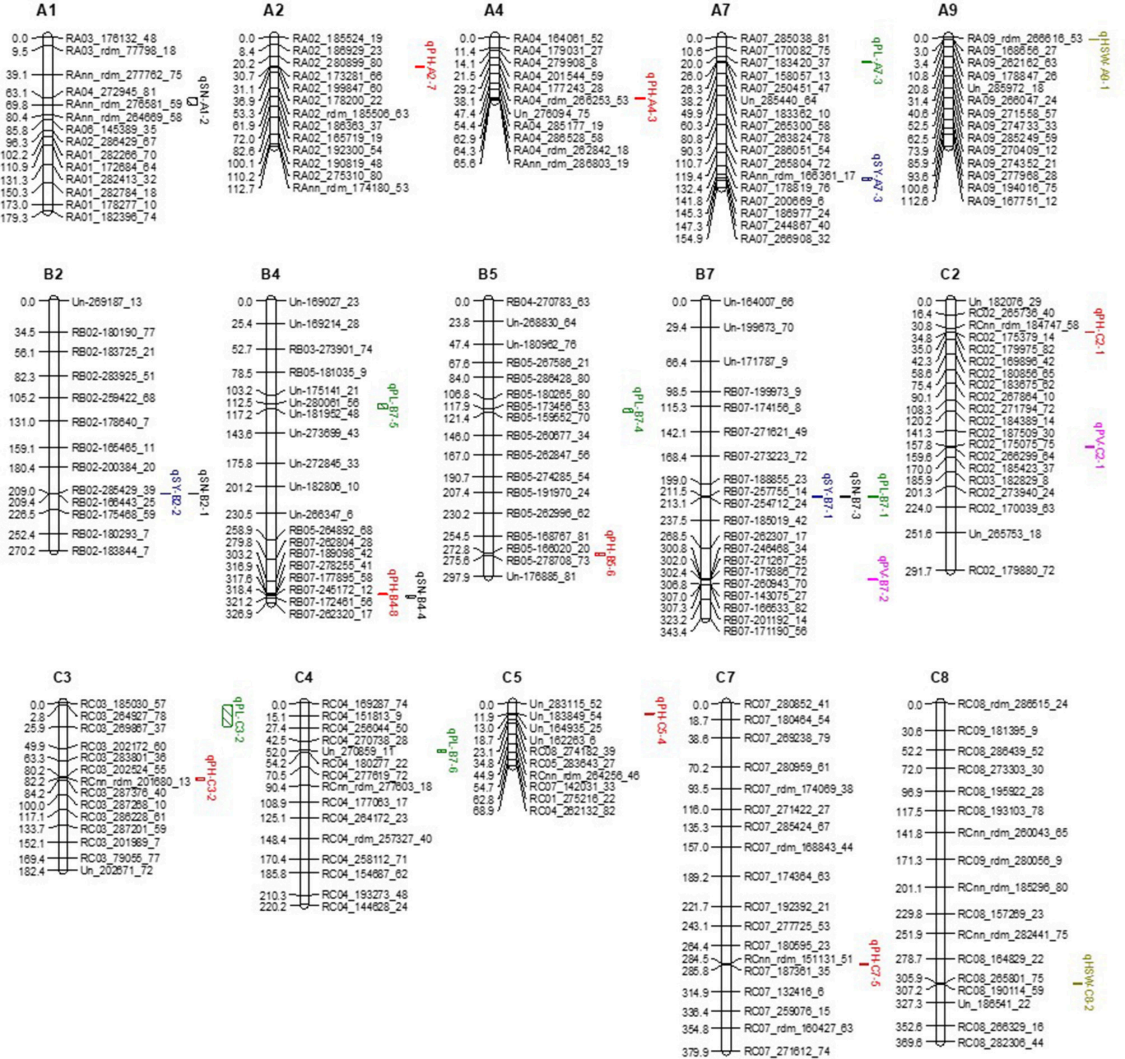
Additive QTL identified for 6 phenotypic traits including seed number, seed yield, pod length, plant height, 1000-seed weight and pollen viability using ICIM method of QTL IciMapping 4.1 on a doubled haploid mapping population derived from allohexaploid *Brassica* hybrid H16-1. QTL for seed number, seed yield, pod length, plant height, 1000-seed weight and pollen viability are black, blue, green, red, brown and pink, respectively.

The genotypic and phenotypic data from two parents and 146 DH lines in the first generation DH population were analyzed to detect additive QTL and A × A QTL. Altogether 25 QTL-peak loci were identified for the 6 phenotypic traits, including 4, 3, 6, 8, 2, and 2 QTL for seed number (*qSN*), seed yield (*qSY*), pod length (*qPL*), plant height (*qPH*), 1000-seed weight (*qTSW*) and pollen viability (*qPV*), respectively (Table 7, Figure 3). Among these 25 additive QTL, one QTL contributed more than 10% phenotypic variance explained (PVE), and 24 QTL each contributed at least 3% PVE (Table 7).

**Table 7:** Additive QTL identified among 6 phenotypic traits including seed number, seed yield, pod length, plant height, 1000-seed weight and pollen viability using ICIM method of QTL IciMapping 4.1 (Wang et al., 2016) in the allohexaploid *Brassica* parents and 146 doubled haploid progenies derived from a hexaploid *Brassica* hybrid H16-1.

**Trait**	**QTL**	**Chr**	**Position (cM)**	**LOD**	**Flanking marker**	**Additive effect**	**PVE (%)**
Seed number	*qSN-B2-1*	B2	209.00	9.46	RB02-285429_39 and RB02-166443_25	21.52	13.17
Seed number	*qSN-A1-2*	A1	69.00	5.05	RA04_272945_81 and RAnn_rdm_276581_59	−15.33	6.69
Seed number	*qSN-B7-3*	B7	212.00	4.19	RB07-257755_14 and RB07-254712_24	13.90	5.48
Seed number	*qSN-B4-4*	B4	319.00	1.59	RB07-245172_12 and RB07-172461_56	8.12	3.71
Seed yield	*qSY-B7-1*	B7	212.00	3.17	RB07-257755_14 and RB07-254712_24	0.04	8.06
Seed yield	*qSY-B2-2*	B2	209.00	2.59	RB02-285429_39 and RB02-166443_25	0.04	6.48
Seed yield	*qSY-A7-3*	A7	147.00	1.52	RA07_186977_24 and RA07_244867_40	−0.03	3.97
Pod length	*qPL-B7-1*	B7	213.00	2.31	RB07-257755_14 and RB07-254712_24	3.28	7.06
Pod length	*qPL-C3-2*	C3	7.00	2.18	RC03_264927_78 and RC03_269867_37	−3.47	7.68
Pod length	*qPL-A7-3*	A7	26.00	2.18	RA07_158057_13 and RA07_250451_47	3.16	6.63
Pod length	*qPL-B7-4*	B5	121.00	2.01	RB05-173456_53 and RB05-159552_70	3.04	6.12
Pod length	*qPL-B7-5*	B4	117.00	1.91	Un-280061_56 and Un-181952_48	2.96	5.82
Pod length	*qPL-B7-6*	C4	53.00	1.69	Un_270859_11 and RC04_180277_22	2.85	5.29
Plant height	*qPH-C2-1*	C2	35.00	1.94	RC02_175379_14 and RC02_179975_82	−4.39	5.89
Plant height	*qPH-C3-2*	C3	83.00	1.86	RCnn_rdm_201680_13 and RC03_287376_40	−4.34	5.67
Plant height	*qPH-A4-3*	A4	64.00	1.83	RA04_286528_58 and RA04_rdm_262842_18	−4.29	5.63
Plant height	*qPH-C5-4*	C5	12.00	1.67	Un_183849_54 and Un_164935_25	−4.08	5.09
Plant height	*qPH-C7-5*	C7	285.00	1.59	RCnn_rdm_151131_51 and RC07_187361_35	4.02	4.90
Plant height	*qPH-B5-6*	B5	273.00	1.51	RB05-166020_20 and RB05-278708_73	−3.90	4.63
Plant height	*qPH-A2-7*	A2	31.00	1.46	RA02_173281_66 and RA02_199847_60	3.83	4.47
Plant height	*qPH-B4-8*	B4	317.00	1.44	RB07-278255_41 and RB07-177895_58	−3.81	4.43
1000-seed weight	*qTSW-A9-1*	A9	3.00	1.55	RA09_168656_27 and RA09_262162_63	−0.04	4.74
1000-seed weight	*qTSW-C8-2*	C8	306.00	1.42	RC08_265801_75 and RC08_190114_59	0.04	4.36
Pollen viability	*qPV-C2-1*	C2	158.00	1.58	RC02_175075_75 and RC02_266299_64	−5.84	4.84
Pollen viability	*qPV-B7-2*	B7	301.00	1.47	RB07-246468_34 and RB07-271267_25	−5.72	4.55

*QTL are designated, for example, as qSN-B2-1, where q = QTL, SN, seed number; B2, chromosome B2; 1, first QTL for this phenotypic trait. Chr, chromosome; LOD, logarithm of odds; PVE, phenotypic variation explained. Positive additive effects indicate that the maternal parent donates the positive allele, whereas negative additive effects indicate that the maternal parent donates the negative allele*.

Four *qSN* QTL related to seed number (SN) were located on chromosomes A1, B2, B4, and B7 (Table 7). Among them, *qSN-B2-1* explained 13.17% PVE with high LOD value (9.46). In total, these 4 QTL explained 29.05% PVE. Three *qSY* QTL related to seed yield (SY) were found on chromosomes A7, B2, and B7. These three QTL contributed 18.51% PVE. Interestingly, *qSN-B2-1* and *qSY-B2-2* were co-located on chromosome B2, which suggests they are controlled by the same gene(s). Eight *qPH* QTL related to plant height (PH) were distributed across chromosomes A2, A4, B4, B5, C2, C3, C5, and C7. The PVE of these 8 QTL ranged from 4.43 to 5.89% with an average of 5.09%. Six *qPL* related to pod length (PL) were located on chromosomes A7, B4, B5, B7, C3, and C4. These 6 QTL altogether represented 38.61% PVE and on average every QTL explained more than 5% PVE for pod length. Three QTL *qSN-B7-3, qSY-B7-1*, and *qPL-B7-1* were co-located on B7 (Table 7, Figure 3), which is a genomic hotspot for these three yield-related phenotypic traits. In addition, 2 *qTSW* related to thousand seed weight (TSW) were located on chromosomes A9 and C8. Two *qPV* related to pollen viability (PV) were located on chromosomes B7 and C2 each explained less than 5% PVE (Table 7, Figure 3).

The QTL for all 6 phenotypic traits were mapped on 15 chromosomes including 5 A-genome chromosomes, 4 B-genome chromosomes and 6 C-genome chromosomes (Figure 3). Four QTL were located on chromosome B7 and 3 on chromosome B4. Chromosomes A7, B2, B5, C2, and C3 each had two QTL, and chromosomes A1, A2, A4, A9, C4, C5, C7, and C8 each had one QTL (Figure 3). Among the 25 QTL controlling 6 phenotypic traits, 12 had positive additive effects and 13 had negative additive effects, and both parents contributed positive alleles. Selection for improvement in these traits is therefore possible in the DH progeny (Table 7, Figure 3).

A total of 62 epistatic (A × A) QTL pairs were distributed on 27 chromosomes for SN (20), SY (23), TSW (17) and PV (2). No A × A QTL were detected for PL and PH. The LOD value of these 62 epistatic QTL pairs ranged from 5.14 to 29.72. Among the 62 epistatic QTL, 44 were intragenomic and 18 were intergenomic. For 44 intragenomic QTL pairs, 33 pairs were located on the same chromosomes in the A, B, and C genomes, and appeared to favor certain chromosomes—for example, 3 were located on each of A1, A3, A4, B7, and C7 (Table S3). Ten pairs were on different chromosomes within the B genome, and one pair was on different chromosomes in the C genome. For the 18 intergenomic QTL pairs, three A × A QTL were identified between the A1 and C1 chromosomes, 5 were found between the A and B genomes and 10 were located between the B and C genomes (Table S3). A1 and C1 also had relatively few mapped QTL (Figure 1).

For PV, two positive A × A QTL were identified with 10.28 and 10.36% PVE, respectively, between C1–C8 and B1–C8 with a common position 330 on C8 (Table S3). The remaining 60 A × A QTL controlling 3 yield-related traits (SN, SY, and TSW) were all negative. The total A × A PVE for SN, SY, TSW, and PV was 56.73, 59.04, 46.55, and 20.49%, respectively (Table S3).

## Discussion

### Selection of SNPs for mapping

From a putative 416,238 SNPs identified from clustering of RAD reads, 329,244 were discarded because they were missing from >8% of DH progeny and 5,015 were discarded due to significant segregation distortion in the DH population. This high rate of loss of SNP markers from DH progeny most likely reflects abnormal chromosome pairing during meiosis which is common in synthetic tetraploid and hexaploid *Brassica* (Udall et al., 2005; Mason et al., 2015). Compared with fertilization and seed set, more abnormal gametes may survive the DH process which will increase the proportion of missing SNPs in DH progeny. Homoeologous nonreciprocal transpositions (HNRTs) may cause a loss of a portion of a chromosome in one of the homoeologous pair (e.g., A1), and a duplication of the genome in the other of the homoeologous pair (e.g., C1) as described by Udall et al. (2005). Our results show very short linkage groups for A1 and C1 (Figure 1), which may reflect this disruption due to HNRTs. Interestingly, we also found three A1-C1 epistatic interactions for phenotypic traits important for survival of this new allohexaploid population (Table S3).

Marker-segregation distortion is common in plants, and could be the result of gene duplication or deletion, transposable elements and meiotic segregation distortion disorders (Li and He, 2014). In order to find stable chromosomal elements in this mapping population, we used only markers that conformed to the expected Mendelian segregation ratio in DH populations of 1:1 (*P* < 0.05) for linkage map construction.

### Linkage map construction and analysis

Previously we reported a framework linkage map of this hexaploid *Brassica* DH mapping population based on 274 SSR markers distributed across 27 chromosomes with a total genetic distance of 3178.8 cM (Yang et al., 2016b). This provided valuable information for further genetic improvement of a new allohexaploid *Brassica* species. However, its use for QTL identification was limited due to low resolution (the average interval between SSR markers ranged from 5.9 to 32.2 cM). Thus, in the present research, we used RAD-seq which is faster, more robust and detects large numbers of SNPs. We then combined these SNPs with previously-mapped SSRs to construct a combined linkage map, and allocated chromosome numbers based on the published physical maps of the A, B, and C genomes (Wang et al., 2011; Liu et al., 2014; Yang et al., 2016a).

The new linkage map was constructed with 7,499 SNPs and 163 SSRs distributed across 27 chromosomes, with a total genetic distance of 5725.19 cM and an average distance between adjacent markers of 0.75 cM. This new high-density map has a longer total genetic distance across the A, B, and C genomes than the original map (Yang et al., 2016b). One possible reason for this is the very low number of B-genome markers in the previous study (Yang et al., 2016b), and irregular distribution of markers, so that more genetic recombination is detected in this high-density map.

Physical mapping was achieved on the progeny of crosses between hexaploid parents, and this enabled us to map several unassigned SNP markers to chromosomes. However, many unassigned SNP markers were in XLGs, notably XLG1 (Figure S1). This phenomenon had also been observed in our framework linkage map (Yang et al., 2016b). These unassigned markers may be caused by relatively frequent homoeologous exchange during abnormal meiotic behavior in hybrid H16-1 and the DH population since synthetic allohexaploid *Brassica* is expected to experience chromosome changes and meiotic chromosome pairing errors during the polyploidisation process (Geng et al., 2013; Yang et al., 2016b). For small XLGs or single unassigned SNP markers, they may be distributed at the extreme ends of chromosomes and distant from other markers (Yang et al., 2016b). These loci may join onto the ends of smaller linkage groups as additional markers are screened in this mapping population. Unassigned SNP markers may also result from very low recombination rates and high mapping distance in some areas of the genome. The unmapped SNPs and six uncertain linkage groups did not prevent the detection of valuable QTL for seed yield and pollen viability. We conclude that this DH population may be selected for improved stability of a future allohexaploid *Brassica* species.

This high-density linkage map contains detailed genomic information about hexaploid *Brassica* that could provide a foundation for a better understanding of the genetic structure of *Brassica* species (Li and He, 2014).

### Collinearity with reference genomes and possible reasons for changes in the genetic map

Approximately 84% of all the mapped SNPs were blasted onto the reference genomes of the three *Brassica* species *B. rapa, B. nigra*, and *B. oleracea*, and 21/27 chromosomes were mapped with high collinearity to the reference genomes (Figure 2) with high rates of common SNPs, which suggests that the segregating allohexaploid population had strong homology to the reference genomes of the parental diploid species (Figure 2). After checking the restriction enzyme sites, we found that the SNP-targeted restriction sites were evenly distributed across the whole genome. Despite this, six chromosomes were not mapped with high confidence and had low rates of common SNPs (B1, B3, B4, B8, C1, and C5).

In a previous study, we observed some abnormal chromosome pairing and segregation during meiosis in F_1_ hybrid H16-1, such as chromosome bridges, laggards and chromosome loss (Geng et al., 2013). The chromosomes which were not mapped with high confidence in this study may have been involved in homoeologous pairing. For example, chromosomes A1 and C1 were notably low in SNPs (Figure 1), and this pair of chromosomes is often involved in homoeologous exchange in synthetic *Brassica* polyploids (Udall et al., 2005; Mason et al., 2015). Variation in genetic distance between SNP markers was often found within chromosomes, indicating that some regions had more recombination than others. We also noticed that in this allohexaploid population, sometimes SNP markers were assigned to different chromosomes instead of the blasted position in the reference genome. For example, RA08_214458_9 and RB03-168918_12 were on A8 and B3 in the *B. rapa* and *B. nigra* reference genomes, but on this allohexaploid linkage map they were located on A2 and B8 respectively. Marker RC02_197838_37, previously located on C2 chromosome in *B. oleracea* reference genome, was assigned to C1 on this allohexaploid map.

The redistribution of SNP markers in the DH population compared with the position on reference genome may also be the result of chromosome rearrangements during meiosis in H16-1, as suggested previously with an SSR-based linkage map (Yang et al., 2016b). The allohexaploid parents of H16-1 were derived from four different *Brassica* species (Geng et al., 2013), which may contribute to abnormal chromosome pairing. However, our discovery of additive QTL for seed yield and pollen viability in this allohexaploid × allohexaploid DH mapping population raises the anticipation that progeny of such crosses may be selected with stable chromosome arrangements in near-hexaploid form.

### Breeding a future allohexaploid *Brassica* species

In this study, “QTL hot-spots” for yield-related traits were found on B2 and on B7 (Table 7). Such QTL hot-spots were also observed in soybean (Zhang et al., 2004), *Brassica juncea* (Ramchiary et al., 2007) and rice (Xiao et al., 1998), and support the conclusion that pleiotropy or closely-linked genes explain the genetic basis of correlated traits (Veldboom and Lee, 1994). Intercrossing among DH progeny in this hexaploid *Brassica* population with complementary QTL for pollen viability and fertility traits will potentially improve the yield and stability of future allohexaploid *Brassica*.

## Availability of supporting data

The data discussed in this publication have been deposited in NCBI's Sequence Read Archive (SRA) database SRA and the accession number is SRP142247 (https://www.ncbi.nlm.nih.gov/sra/SRP142247).

## Author contributions

SY and SC are equal first contributing authors to this paper. The conceptualization and funding support for this project were generated by WZ, WAC, SC, JM, and GY. The research component was conducted by SY, SC, LL, KZ, and YY. The analysis was conducted by SY, SC, and RAG. The manuscript was written and revised by SY, SC, WAC, and WZ with input from all other co-authors.

### Conflict of interest statement

The authors declare that the research was conducted in the absence of any commercial or financial relationships that could be construed as a potential conflict of interest.

## Supplementary material

Table S1 SNPs and Indels variation types and their distribution in the allohexaploid *Brassica* parents and 146 doubled haploid progenies. P1 is maternal line 7H170-1, P2 is paternal line Y54-2, H1 is the hybridization product (H16-1) between two parents while SY003 to SY192 are 146 doubled haploid progenies derived from H16-1.

Table S2 SNPs matrix for linkage map constructing and their chromosome location. P1 is maternal line 7H170-1, P2 is paternal line Y54-2 while SY003 to SY192 are 146 doubled haploid progenies derived from H16-1.

Table S3 Epistatic QTL pairs identified among 6 phenotypic traits including seed number, seed yield, pod length, plant height, 1000-seed weight and pollen viability ICIM-ADD method of QTL IciMapping 4.1 in a double haploid population derived from a hexaploid *Brassica* hybrid H16-1. “Intra” means intra-genomic QTL pairs while “Inter” means inter-genomic QTL pairs. “S” means same chromosome, “D” means same genome but different chromosomes. “A–B,” “A–C,” “B–C” means one QTL located at the first genome and the other QTL located at the lateral genome. “Chr1” and “Chr2” mean the chromosome of the first and second additive QTL. “Pos1” and “pos2” mean the exact position on the chromosome of each additive QTL. “PVE” means phenotypic variation explained of each epistatic QTL.

Table S4 Summary of possible loss of chromosome(s) or chromosome segment(s) of 146 DH hexaploid *Brassica* plants. If more than 80% of SNPs are missing on a particular chromosome, this is shown as “loss of chromosome.” If more than 20% but less than 80% of SNPs are missing as a contiguous segment on a chromosome, this is shown as “loss of chromosome segment.”

Figure S1 562 SNP and SSR markers which failed to be integrated into the high-density linkage map. Among them, there were 66 unlinked single loci and 48 extra linkage groups (XLG) including 3 linkage groups with more than 50 markers (XLG1 to 3), 7 linkage groups with 6 to 50 markers (XLG 4 to 14), five quintuplets, six quadruplets, seven triplets and 16 duplets.
