# Supplementary figures and images for: A High-Density Genetic Map of an Allohexaploid Brassica Doubled Haploid Population Reveals Quantitative Trait Loci for Pollen Viability and Fertility
Source: Front Plant Sci. 2018 Aug 28;9:1161. doi: 10.3389/fpls.2018.01161 (PMC6123574; doi:10.3389/fpls.2018.01161)

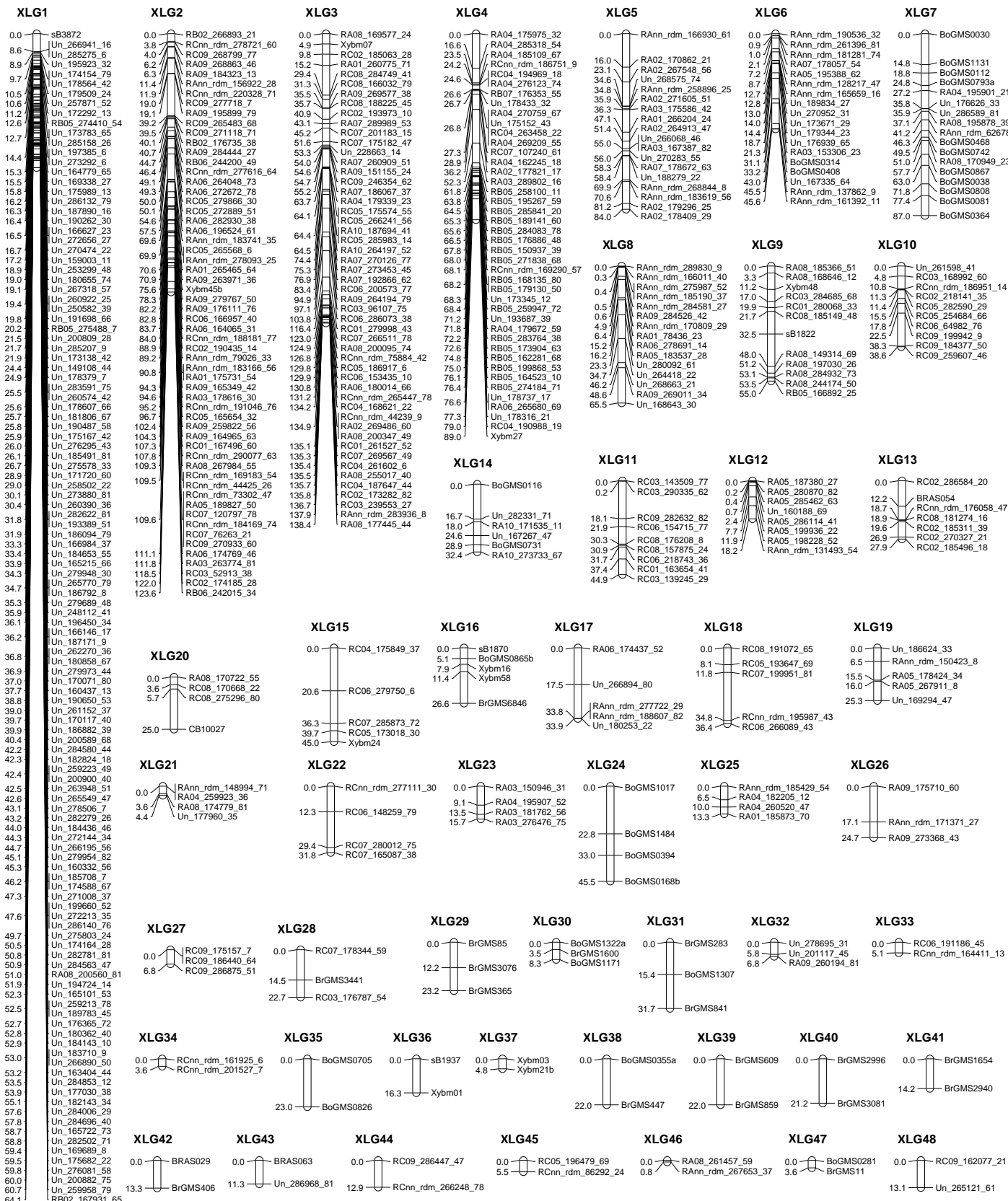

Supplement: Figure S1 — 562 SNP and SSR markers which failed to be integrated into the high-density linkage map. Among them, there were 66 unlinked single loci and 48 extra linkage groups (XLG) including 3 linkage groups with more than 50 markers (XLG1 to 3), 7 linkage groups with 6 to 50 markers (XLG 4 to 14), five quintuplets, six quadruplets, seven triplets and 16 duplets. [file Image_1.pdf]

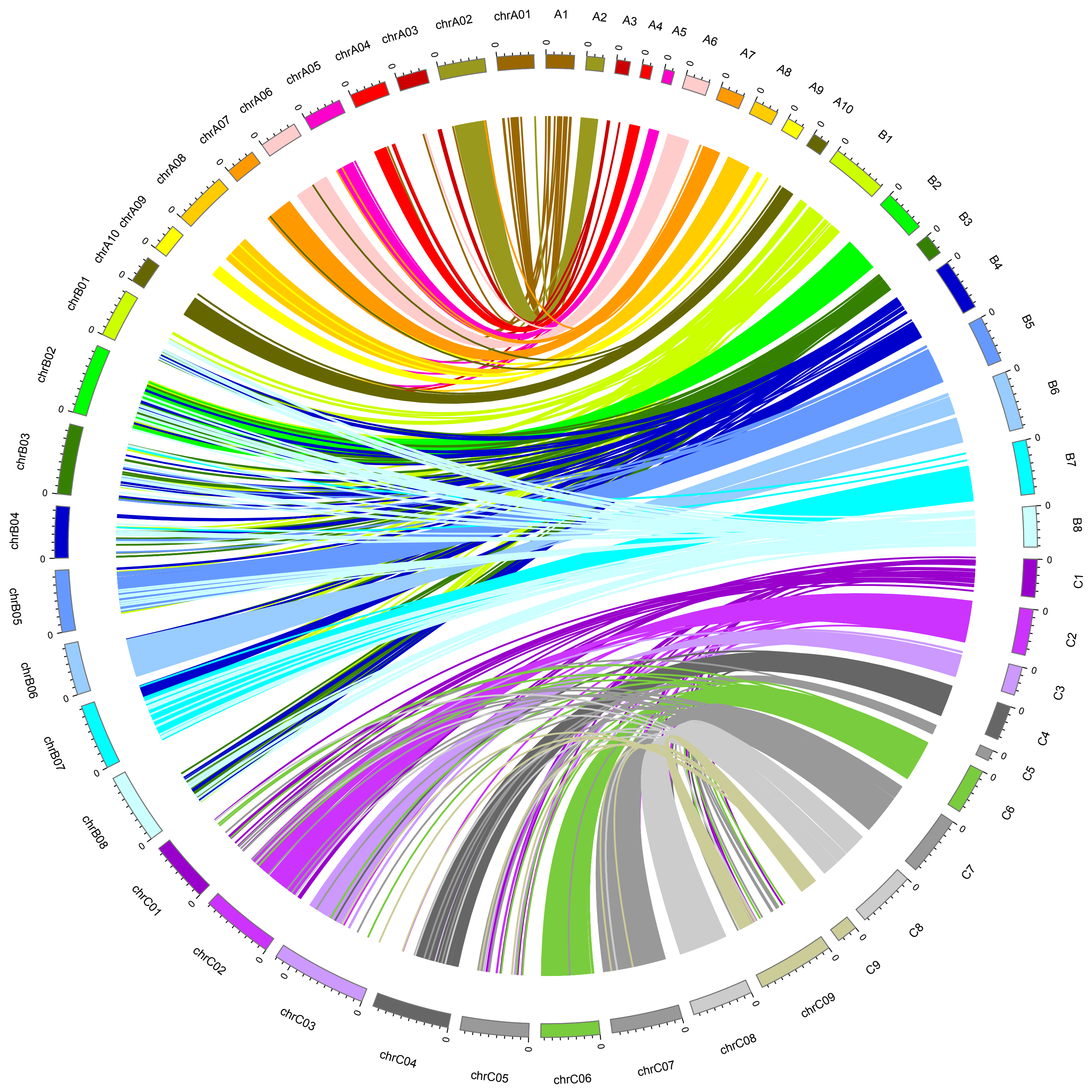

Supplement: Supplementary file 3 [file Data_Sheet_1.PDF]
